# Does Emerging Carbapenem Resistance in *Acinetobacter baumannii* Increase the Case Fatality Rate? Systematic Review and Meta-Analysis

**DOI:** 10.3390/idr15050055

**Published:** 2023-09-27

**Authors:** Jale Boral, Fatihan Pınarlık, Güz Ekinci, Füsun Can, Önder Ergönül

**Affiliations:** 1 Graduate School of Health Sciences, Koç University, Istanbul 34010, Türkiye; jboral20@ku.edu.tr (J.B.);; 2 Koç University İşBank Center for Infectious Diseases, Koç University Hospital (KUISCID), Istanbul 34010, Türkiye; fucan@ku.edu.tr; 3 Department of Medical Microbiology, School of Medicine, Koç University, Istanbul 34010, Türkiye; 4 Department of Infectious Diseases and Clinical Microbiology, School of Medicine, Koç University, Istanbul 34010, Türkiye

**Keywords:** *Acinetobacter baumannii*, systematic review, carbapenem resistance, mortality rate, molecular epidemiology

## Abstract

Background: In the era of rising carbapenem resistance, we aimed to investigate the change in mortality rate and positivity of carbapenemase genes in *Acinetobacter baumannii*. Methods: Preferred Reporting Items for Systematic Review (PRISMA) guidelines were adopted in this systematic review. Our literature search included the Cochrane Library, Pubmed, Scopus, Web of Science, Medline, Tubitak TR Dizin, and Harman databases for studies dating back from 2003 to 2023 reporting bloodstream *A. baumannii* infections in Türkiye. A simple linear regression model was used to determine the association between resistance, mortality, and time. Results: A total of 1717 studies were identified through a literature search, and 21 articles were selected based on the availability of the data regarding mortality and resistance rate (four articles) or the molecular epidemiology of carbapenem-resistant *A. baumannii* (17 articles) in Türkiye. From 2007 to 2018, the carbapenem resistance rate increased (*p* = 0.025). The OXA-23 and OXA-58 positivities were inversely correlated (*p* = 0.025). Conclusions: Despite the emergence of carbapenem resistance, mortality did not increase in parallel, which may be due to improved medical advancements or the fitness cost of bacteria upon prolonged antimicrobial exposure. Therefore, we suggest further global research with the foresight to assess clonal relatedness that might affect the carbapenem resistance rate.

## 1. Introduction

*Acinetobacter baumannii* is a non-fermenting gram-negative pathogen, playing a major role in nosocomial infections, especially in the intensive care units [1]. *A. baumannii* is a pathogen of concern due to its extensive ability to survive on dry and abiotic surfaces for at least 20 days, tolerate desiccation as well as high temperatures, and gain resistance to many available antimicrobial treatments, especially carbapenems such as meropenem and imipenem [2,3,4]. As a leading pathogen to cause many hospital-acquired infections such as ventilator-associated pneumonia, urinary tract infections, meningitis, and bloodstream infections [5], it remains an outstanding challenge to diagnose and treat *A. baumannii* infections with an appropriate treatment due to its ability to survive in various environments and its capability of gaining resistance to multiple antibiotics [6]. The emergence of carbapenem-resistant *Acinetobacter baumannii* (CRAB) and its association with high morbidity and mortality [7] have created a need for screening the resistance levels in contrast with the associated mortality.

In 2017, the World Health Organization listed carbapenem-resistant *A. baumannii* as the first priority pathogen of concern for research and development of a novel antibiotic therapy due to its high morbidity and mortality rates [8]. While emerging antimicrobial resistance, especially in *A. baumannii*, is a constant threat, the introduction of the COVID-19 pandemic as of 2020 has resulted in an increase in the rates of *A. baumannii* infections, co-infections, and case fatality rates compared to the pre-pandemic era, boosting the importance of antimicrobial resistance and mortality surveillance [9].

In 2007, the carbapenem resistance rate in *A. baumannii* was reported as 67.5% [10], while in 2018, it was reported to be 96.5% in Türkiye [11]. This drastic increase in carbapenem resistance over the last decade has shifted our focus to its association with mortality rates. In this systematic review and meta-analysis, we aim to illuminate the association of mortality rate trends caused by multidrug-resistant *A. baumannii* bloodstream infections with the increasing carbapenem resistance over the past 15 years. Our secondary aim is to evaluate the positivity and types of carbapenem-hydrolyzing carbapenemase genes in CRAB in Türkiye to further demonstrate an epidemiological evaluation on a molecular basis. In doing so, we aimed to highlight the dominant carbapenemases produced by CRAB in Türkiye.

## 2. Materials and Methods

### 2.1. Study Design

The systematic review was performed using Preferred Reporting Items for Systematic Review (PRISMA) guidelines. This study is registered with PROSPERO under the ID number CRD42023284086 as of 4 April 2023.

### 2.2. Existing Reviews

Two authors (J.B. and F.P.) searched independently for the previously published systematic reviews and meta-analyses for similar reviews. Online research included the Cochrane Library, Pubmed, Scopus, Web of Science, Medline, Tubitak TR Dizin, and Harman databases. No similar review was conducted.

### 2.3. Search Strategy

In order to collect retrospective studies of 15 years on the mortality rate of *A. baumannii* infections based on its carbapenem resistance and case fatality rates, comprehensive online research was performed through the Cochrane Library, Pubmed, Scopus, Web of Science, Medline, Tubitak TR Dizin, and Harman databases dating back from 2003 to 2023. Tubitak TR Dizin and Harman databases were especially included to enhance comprehensive research within Türkiye, as these databases include the majority of studies published in Turkish in Türkiye. The studies were limited to infections on humans in Türkiye, and no language restrictions were put on the search or eligibility criteria. To obtain a specified set of data for further scanning, keywords used were the following: “Acinetobacter baumannii”, “resistance”, “bloodstream”, or “blood circulation”, or “blood flow”, or “bacteremia”, “nosocomial”, “intensive care unit”, “case fatality rate”, or “mortality”, or “death”. The articles that were selected for the retrieval step were screened for their references as additional eligible study candidates. The last database search was performed on 5 January 2023.

### 2.4. Selection Criteria

Records were screened and assessed independently by two authors (J.B. and F.P.). Abstracts of the selected records were assessed for their eligibility to be screened further by the authors J.B., F.P., and G.E., independently. Prior full text screening was performed after the article was approved by at least two independent authors as a potential article for inclusion. Records that were found to be potentially inclusive and relevant were fully screened for their references as a potential source for new article inclusions. We included descriptive studies that presented proper information on the years covered during the isolate collection and attributed mortality rates specific to the *A. baumannii* bloodstream infections and resistance rates to carbapenem. We excluded studies involving pediatric patients, and the sample size from each study was limited to a minimum of 40. Selected studies that did not provide data on standardized definitions for AMR and that did not refer to standardized guidelines such as those of the Clinical and Laboratory Standards Institute (CLSI) or the European Committee on Antimicrobial Susceptibility Testing (EUCAST) on reporting susceptibility profiles were further discarded. Data extraction from the selected articles was performed by J.B. and F.P., which was further cross-confirmed by G.E.

### 2.5. Quality Assessment

The Newcastle–Ottawa Quality Assessment Scale (NOS) for Cohort Studies [12] was selected to evaluate the bias of selected articles by the authors J.B. and F.P. NOS was selected based on its suitability to assess the quality of non-randomized studies in systematic review and meta-analysis studies. All selected articles scored 7 points, which is considered a high-quality study (Table 1). Three out of 4 articles [11,13,14] lost score points due to being retrospective studies, and the remaining article lost a score point due to the poor comparability of cohorts in the design and analysis of the study [10].

### 2.6. Data Synthesis and Analysis

During data synthesis, we focused on the association between time, resistance, and mortality. For studies that covered multiple years but did not report their results separately, the results were assumed to be reporting the latest year of patient recruitment. Carbapenem resistance rates and mortality rates obtained from studies published in the same year were averaged. However, the studies that reported their results for different years separately were analyzed as independent studies. Additionally, the associations between carbapenemase genes and their association with time were investigated. GraphPad Prism 9.5.1 (GraphPad Software, San Diego, CA, USA) was used to illustrate the time-dependent change in mortality, resistance, and the prevalence of carbapenemase genes. A forest plot was generated to illustrate and analyze mortality data for patients with carbapenem-resistant *A. baumannii* (CRAB) versus carbapenem-susceptible *A. baumannii* infections. In order to test the association between time, resistance, and mortality, we used a simple linear regression model. All the analyses were conducted via IBM SPSS Statistics Version 28.0 (IBM Corp., Armonk, NY, USA), and a *p*-value less than 0.05 was considered statistically significant.

For molecular screening, we focused on carbapenemase positivity in carbapenem-resistant *A. baumannii* isolates based on PCR results. The PCR primer sequences used for carbapenemase screening were checked for compatibility. The same set of studies were screened for clonality of CRAB isolates based on pulsed field gel electrophoresis (PFGE) and repetitive element sequence-based PCR (rep-PCR) results. Modifications made to the protocols of the PFGE and the rep-PCR methods were screened for compatibility to assess clonality. Both methods were found adequate and comparable to assess the clonality of isolates originating from the same centers [15,16].

## 3. Results

**Figure 1:**
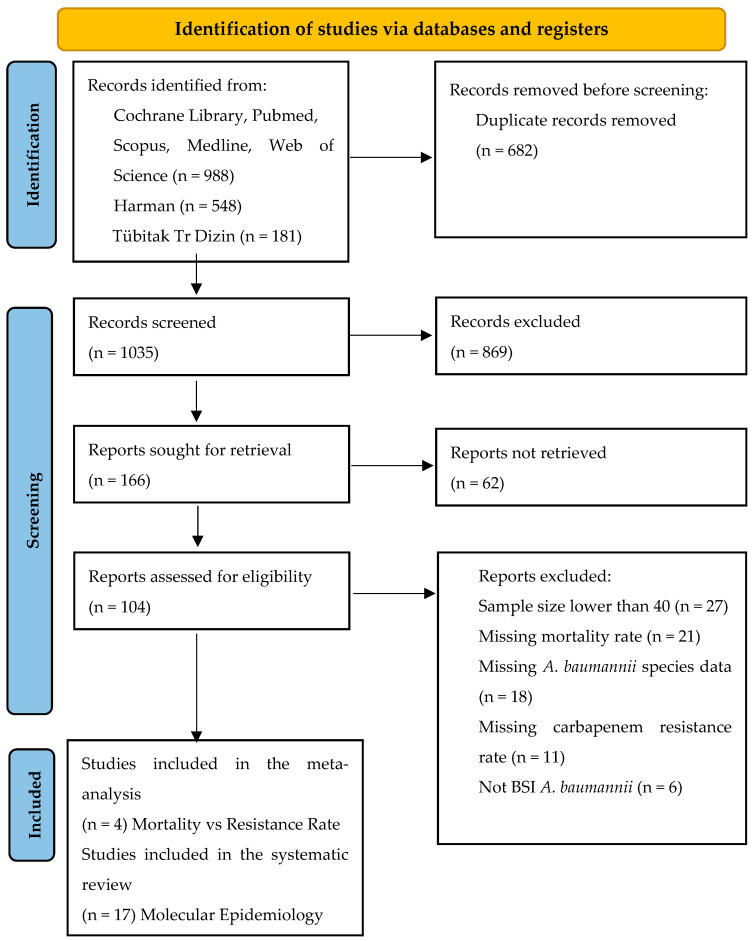
PRISMA flow diagram study selection process.

**Figure 2:**
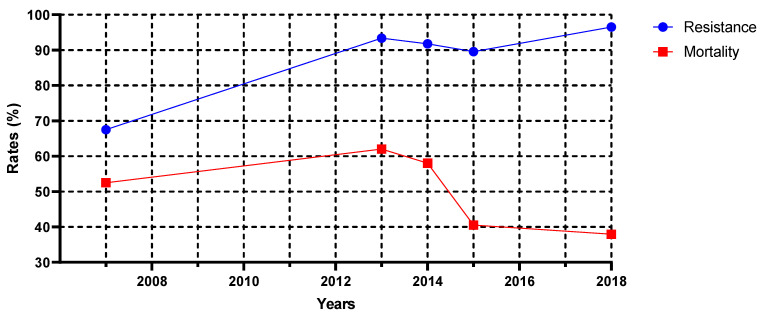
Corresponding carbapenem resistance and case fatality rates of *A. baumannii* between the years of 2007 and 2018.

In total, 1717 studies were identified through the database search from Cochrane, Pubmed, Scopus, Medline, Web of Science (N = 988), Harman (N = 548), and TR Dizin (N = 181). A total of 682 duplicate studies were removed. Out of 1035 studies, 869 were excluded due to being a mismatch for the determined criteria and missing relevant data, while 166 studies were found suitable and analyzed further in full text by authors J.B., F.P., and G.E. (Figure 1). Twenty-one out of 166 articles were found eligible for further systematic review and meta-analysis of the study. Two main categories were formed. Four out of 21 articles were selected for the first category of analysis, which was to investigate the association between the case fatality rate and the carbapenem resistance rate of *A. baumannii* infections. The second category has 17 articles, which were formed to further assess the molecular epidemiology of carbapenemase positivity amongst carbapenem-resistant *A. baumannii* isolates in Türkiye [17,18,19,20,21,22,23,24,25,26,27,28,29,30,31,32,33] (Table 2). A total of 1456 patients with *A. baumannii* bloodstream infections were included in this study, dating from 2007 to 2018. Overall, four studies selected for the analyses all provided attributed mortality, morbidity, and carbapenem resistance rates of *A. baumannii* isolates [10,11,13,14] (Figure 2). Twelve out of 17 studies selected for molecular epidemiology surveillance provided data on the clonal relatedness of the bacterial isolates used in the study by means of PFGE and rep-PCR experiments. Additionally, all 17 of these studies provided results for three carbapenemase genes: bla_OXA-23_, bla_OXA-58_, and bla_OXA-24_. All selected studies for molecular surveillance stated 100% positivity for bla_OXA-51_, which is also known as the intrinsic oxacillinase for *A. baumannii* species found in the chromosome [34].

**Figure 3:**
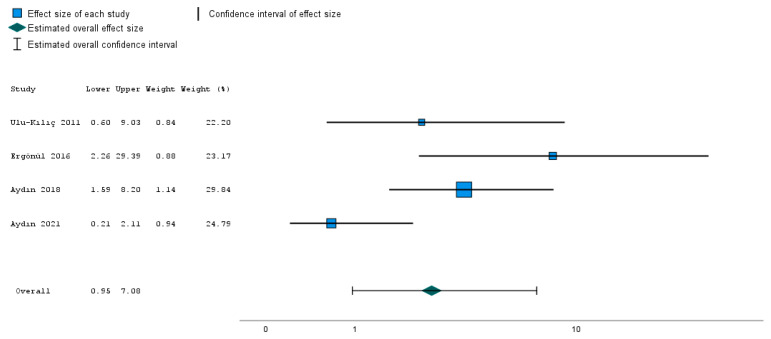
Forest plot of mortality in patients with carbapenem-resistant *A. baumannii* (CRAB) versus carbapenem-susceptible *A. baumannii* infections. OR: odds ratio [10,11,13,14].

Our analysis suggests that carbapenem resistance rates were increasing between 2007 and 2018 (*p* = 0.025). However, there was no statistical significance between the time and the mortality (*p* = 0.384) as well as the carbapenem resistance rates and the mortality (*p* = 0.808). Additionally, the coexistence of bla_OXA-23_ and bla_OXA-58_ was not observed; on the contrary, their positivity rates were inversely correlated (*p* = 0.025). However, no correlation was found to be statistically significant (*p* > 0.05) between other resistance genes and time. Eventually, we adopted a random effects model and used the odds ratio for evaluation of the effect size. Although two of the selected articles showed increased mortality in the carbapenem-resistant group compared to the infections with carbapenem-susceptible *A. baumannii*, the overall result was not statistically significant for the effect of carbapenem resistance on the case fatality rate (Figure 3).

## 4. Discussion

Bloodstream infections caused by *A. baumannii* remain as major threat to healthcare [35]. In this systematic review and meta-analysis, we screened a total of 1456 patients with carbapenem-resistant *A. baumannii* bloodstream infections within the years of 2007 to 2018 from the published literature between 2003 and 2023. We selected four articles for the meta-analysis of the *A. baumannii* BSI mortality rate in response to an increasing carbapenem resistance rate (Figure 1). For the evaluation on a molecular epidemiology basis, 17 articles were selected for the systematic review on the diversity and distribution of carbapenemase genes encoded by CRAB in Türkiye.

Since 2009, saturation in CRAB levels has been observed, with the carbapenem resistance rate being reported at around 90%. Our study investigated the correlation between carbapenem resistance rate and case fatality rate in *A. baumannii* infections. Despite a sustained carbapenem resistance rate of 90% over 10 years, the case fatality rate has not increased in parallel, and a simple linear regression model has not suggested statistical significance (*R*^2^ = 0.02294). Although there are previously published studies that have found a statistically significant association between antimicrobial resistance rates and case fatality rates [36,37], our findings did not result in the anticipated association.

One of the potential reasons for this may be stated as increased AMR and prolonged antimicrobial exposure, which come with a fitness cost to bacteria such as reduced virulence and altered pathogenesis [38,39]. In previously published studies, it has been observed that *A. baumannii* has diminished its biofilm formation ability, in vivo dissemination, and surface motility upon conferring colistin resistance [40,41]. Although these studies evaluate this alteration in virulence levels based on colistin exposure and colistin resistance rather than carbapenem, colistin use within the past two decades has increased in the peak era of carbapenem resistance; hence, it would be fruitful to discuss the biological cost of these two antibiotics together with *A. baumannii* [42]. Adaptation of *A. baumannii* to colistin exposure can also be demonstrated by checking resistance values in Türkiye over the past decade, where both carbapenem and colistin are actively being used. While the carbapenem resistance rate in *A. baumannii* has been over 90% since 2015, colistin resistance has only increased from 2.1% to 7% from 2015 to 2018, respectively, despite colistin being widely used over the past decade in Türkiye [11,14]. This may be due to a fitness cost in *A. baumannii*, where it cannot afford to carry colistin resistance and gets rid of it continuously. Hence, colistin exposure to CRAB should be considered not only in terms of resistance but also virulence. On the contrary to previously presented literature [40,41] other studies suggest that colistin resistance does not necessarily cause loss of virulence in *A. baumannii*, as it may enhance and preserve its virulence factors to tolerate colistin rather than conferring resistance to it [43,44]. Eventually, although AMR rates are constantly high for *A. baumannii*, colistin resistance not increasing predominantly over time and loss of virulence upon adaptation to prolonged antimicrobial exposure may be a potential explanation for the case fatality rate remaining statistically unchanged. This highlights the importance of focusing on the research and development of antivirulence therapies, as well as the research of novel antimicrobial agents and targets.

In this study, we have concluded that bla_OXA-23_ and bla_OXA-58_ are the most dominant oxacillinase types produced by CRAB in Türkiye. Both bla_OXA-23_ and bla_OXA-58_ are carbapenem-hydrolyzing plasmid-encoded class D β-lactamases for *A. baumannii* which were one of the earliest carbapenemases to be reported [45]. Meanwhile, no positivity for bla_oxa-24_ was detected, despite 16 out of 17 selected studies including this oxacillinase in their carbapenemase screening tests for CRAB, while neighboring countries have several studies reporting bla_oxa-24_ positivity in CRAB [27,46]. Bla_OXA-23_-producing *A. baumannii* was reported from Bulgaria, China, Brazil, French Polynesia, Iraq, and Afghanistan, whilst bla_OXA-58_-producing *A. baumannii* was mainly reported from Italy, France, Germany, and Japan [47,48,49,50]. Although bla_OXA-23_ is a plasmid-encoded oxacillinase, a study from 2021 has reported the presence of bla_OXA-23_ both in the chromosome and the plasmid of *A. baumannii*, which had little effect on the susceptibility to carbapenem but altered the fitness and virulence of *A. baumannii* [51]. This evolved behavior confers a potential explanation for the statistically indifferent case fatality rate amongst years of MDR. Moreover, our findings show an inverse correlation (*p* = 0.025) for the coexistence of bla_OXA-23_ and bla_OXA-58_ genes, which should be studied further on the basis of bacterial fitness and its impact on virulence. Twelve out of 17 of our selected articles for molecular surveillance have reported the clonal relatedness of isolates by performing PFGE and rep-PCR methods due to the isolates having similar patterns of antimicrobial susceptibility. Although PFGE is reported to have stronger discriminatory power compared to REP-PCR, isolates coming from the same center were found to be suitable to assess with REP-PCR or PFGE [52,53]. Some studies have reported high clonality amongst CRAB samples. The highest clonality was reported as 109 samples belonging to two subgroups [21], followed by another study [23] reporting 62 samples belonging to four subgroups, respectively. Although carbapenem resistance is sustained at high rates above 90%, most of these tested isolates are clonally related. Hence, the high clonality amongst CRAB samples with nosocomial origin may be misleading on the resistance rate evaluation and carbapenemase gene positivity due to the high number of resistant strains belonging to the same subgroups, with a hint at probable outbreaks. Thus, the importance of infection prevention and infection control is highlighted for the detection, containment, and further elimination of such silent outbreaks.

Enhanced medical advancements and scientific innovations in the healthcare industry and appropriate use of antibiotics may also be a potential explanation for the case fatality rate remaining unaffected by the increasing carbapenem resistance rate [54]. Since 2010, rapid identification of bacterial species and susceptibility profiles, as well as rapid detection of antimicrobial resistance genes with high sensitivity and specificity scores, have predominantly decreased the duration of diagnosis and decision on appropriate antimicrobial therapy compared to the early 2000s, enabling healthcare workers to act promptly and accurately at the early onset of an infection [55,56]. Having said that, early diagnostic markers for bacterial infections are also in practice, such as checking procalcitonin (PCT), C-reactive protein (CRP), and interleukin-6 (Il-6) levels [57] to detect a bacterial infection at an early stage and to assess the prognosis of the disease, whilst continuous updates on diagnostic guidelines are being well received in the era of dynamic knowledge exchange and information access [58]. The complementary benefits of these advancements may have led to more focused, customized, and effective therapies compared to the early 2000s, thus affecting the case fatality rate on a downgrading trend.

In addition to medical advancements on rapid and early detection of the disease and its causative pathogen, a study from 2022 recently evaluated their self-developed method of using enhanced detection systems for healthcare-associated transmission (EDS-HAT) to detect silent outbreaks and identify routes of transmissions with this novel method, which combines whole genome sequencing (WGS) and machine learning (ML) while also highlighting the demand for prompt surveillance and action not only upon diagnosis but also upon healthcare-associated transmissions [59]. Although this newly introduced method appears to be more efficient in detecting outbreaks earlier compared to infection prevention and control measures, recent studies report that regular PPE training and hand hygiene scoring of healthcare workers, environmental screening, clonality surveillance of samples, and modified cleaning procedures are still highly effective in the containment and elimination of nosocomial CRAB infections when applied regularly and in compliance [9,60,61]. Moreover, pulsed-field gel electrophoresis remains the gold standard for surveillance of clonality amongst bacterial isolates, and the application of WGS can be challenging to sustain in terms of costs, standardization of protocols, quality assurance, as well as the interpretation of the results [62]. An important take-home message based on infection prevention and control measures can be stated as the customization of such measures based on outbreaks, especially for nosocomial pathogens such as *Acinetobacter baumannii*, *Staphylococcus aureus*, *Pseudomonas aeruginosa*, *Klebsiella pneumoniae*, and *Escherichia coli* [63]. Eventually, this systematic review and meta-analysis highlights the importance of regularly updated guidelines on IPC measures and antimicrobial stewardship programs (ASP) in compliance with communicable reporting systems amongst hospitals, not only for Türkiye, but for co-affected neighboring countries with the same nosocomial pathogens and similar antimicrobial susceptibility patterns [64]. In the context of emerging carbapenem resistance and a lack of suitable antibiotics [65], appropriate IPC measures can suppress the ever-increasing mortality rates by preventing hospital outbreaks.

### Limitations

Several limitations were observed in our systematic review and meta-analysis. Many studies that provided carbapenem resistance rate data and case fatality rate data did not provide data on the attributed mortality for carbapenem-resistant *A. baumannii* infections. Eventually, we had to discard those studies, which lowered the number of selected studies to evaluate, hence the number of cases. Another limitation would be stated as not covering the era of the COVID-19 pandemic within the selected studies. For further research, we suggest that the association between emerging carbapenem resistance and the case fatality rate be comparatively studied for the pre-pandemic and pandemic eras, involving studies from different hospitals and/or study groups to assess the impact of the pandemic upon the emerging antimicrobial resistance and case fatality rate. Moreover, we would like to suggest further studies to report study-specific mortality analysis (i.e., provide sites of infection) to enable an attributed analysis. Eventually, only 12 out of 17 articles provided surveillance on the clonal relatedness of bacterial isolates that shared similar antibiotic susceptibility profiles and carbapenemase positivity.

## 5. Conclusions

Despite the emergence of carbapenem resistance, mortality did not increase in parallel over the past fifteen years, which may be due to improved medical advancements or the fitness cost of bacteria upon prolonged antimicrobial exposure. Therefore, we suggest further global research for a broad scale evaluation and analysis with the foresight to assess clonal relatedness that might affect the carbapenem resistance rate.

## Figures and Tables

**Table 1:** Newcastle–Ottawa score analysis of selected articles. Asterisk (*) defines the positive score obtained from the assessment.

	Ulu Kilic, 2011 [10]	Ergonul, 2016 [13]	Aydin, 2018 [14]	Aydin, 2021 [11]
**Selection Q1** Representativeness of the exposed cohort	*	*	*	*
**Selection Q2** Selection of the non-exposed cohort	*	*	*	*
**Selection Q3** Ascertainment of the exposure	*	*	*	*
**Selection Q4** Demonstration that the outcome of interest was not present at the start of the study	*			
**Comparability Q1** Comparability of cohorts on the basis of design or analysis		*	*	*
**Outcome Q1** Assessment of outcome	*	*	*	*
**Outcome Q2** Follow up long enough for outcomes to occur	*	*	*	*
**Outcome Q3** Adequacy of follow-up of cohorts	*	*	*	*
**Total Score**	**7**	**7**	**7**	**7**

**Table 2:** Presence of OXA-23, OXA-24, and OXA-58 and clonality assessment in *A. baumannii* isolates between 2006 and 2019. (NA: not applicable).

Study	Years Covered	Isolate Number	OXA-23 (%)	OXA-24 (%)	OXA-58 (%)	Clonality (PFGE/repPCR)	Number of Clonal Subgroups
[17]	2006	145	0.0	0.0	79.0	NA	NA
[18]	2010	100	31.0	0.0	23.0	62	13
[19]	2011	834	74.4	0.0	17.3	NA	NA
[20]	2011	201	91.5	1.0	3.5	NA	NA
[21]	2012	109	88.0	0.0	0.0	109	2
[22]	2012	105	46.6	0.0	53.3	49	32
[23]	2012	62	100.0	-	0.0	62	4
[24]	2012	172	96.0	0.0	3.0	172	88
[25]	2013	52	100	0.0	0.0	NA	NA
[26]	2013	79	89.9	0.0	0.0	79	10
[27]	2015	112	67.0	4.5	6.2	112	23
[28]	2015	69	94.2	0.0	0.0	56	7
[29]	2015	96	100	0.0	0.0	NA	NA
[30]	2015	70	100	0.0	0.0	70	22
[31]	2016	177	100	0.0	28.2	175	16
[32]	2019	44	63.6	0.0	0.0	NA	NA
[33]	2022	74	35.1	0.0	43.2	55	6

## Data Availability

Not applicable.
